# Retrospective analysis of the bleeding risk induced by oral antiplatelet drugs during radiotherapy

**DOI:** 10.1097/MD.0000000000024580

**Published:** 2021-02-12

**Authors:** Dan Xi, Wenjie Jiang, Yingjie Shao, Xing Song, Yuan Chen, Mengjiao Liu, Wendong Gu, Qilin Li

**Affiliations:** Department of Radiation Oncology, The Third Affiliated Hospital of Soochow University, Jiangsu Changzhou, China.

**Keywords:** antiplatelet drugs, PSM, radiotherapy, thrombocytopenia

## Abstract

We conducted this retrospective analysis to assess whether oral antiplatelet drugs (APDs) during radiotherapy increase bleeding risk.

Patients who underwent radiotherapy for esophageal cancer (EC) in the Third Affiliated Hospital of Soochow University from January 2015 to December 2019 were screened. After the differences in clinical parameters were eliminated by a propensity-score matched (PSM) analysis at a 1:1 ratio, the thrombocytopenia, consumption of platelet-increasing drugs, suspension of radiotherapy, and bleeding in patients taking APDs were compared with those in the control group.

A total of 986 patients were included in the original dataset. Of these, 34 patients took APDs during radiotherapy. After matching, the APD and control groups each retained 31 patients. There was no significant difference in platelet concentrations between the two groups before radiotherapy (*P* = .524). The lowest platelet concentration during radiotherapy in the APD group was significantly lower (*P* = .033). The consumption of platelet-increasing drugs in the APD group was higher than that in the control group (*P* *<* .05). However, there was no significant difference in the average number of days of radiotherapy suspension because of thrombocytopenia (*P* = .933) and no significant difference in the incidence of bleeding between the two groups (*P* = .605).

Oral APDs during radiotherapy lead to a further decrease in platelet concentration, but timely and adequate application of platelet-increasing drugs can avoid the increased risk of bleeding and the reduced efficacy of radiotherapy.

## 1 Introduction

Radiotherapy is currently an effective method for the treatment of various malignant tumors.^[1,2]^ However, while radiotherapy kills tumor cells, it also affects normal tissue cells of the body.^[3,4]^ Studies have shown that myelosuppression is the most common side effect of radiotherapy for malignant tumors,^[3]^ which is manifested as anemia, leukopenia, and thrombocytopenia.^[5]^ Severe neutropenia and thrombocytopenia can cause infection or bleeding, and often lead to the suspension or even termination of radiotherapy.^[5]^ Therefore, effective prevention and treatment of myelosuppression are the key to ensuring the treatment effect and improving the prognosis in patients with malignant tumors.

The main patients with malignant tumors are elderly individuals, who are also a high-risk population for cardiovascular and cerebrovascular diseases.^[6]^ In clinical practice, we encountered elderly patients with malignant tumors with cardiovascular and cerebrovascular diseases, some of whom took long-term antiplatelet drugs (APDs), such as aspirin, ticlopidine, and clopidogrel.^[7,8]^ Thrombocytopenia is one of the common side effects of these drugs. Although the manufacturers’ instructions claim that the incidence of thrombocytopenia caused by medication is <1/100, we have encountered some patients who continued to take APDs during radiotherapy and had refractory thrombocytopenia. There is no research to explore whether oral APDs during radiotherapy aggravate thrombocytopenia. To resolve this confusion, we retrospectively analyzed the clinical data of patients who continued to take APDs during radiotherapy in our hospital.

To obtain a large enough sample size and to avoid the heterogeneity caused by different types of tumors, we chose esophageal cancer (EC), a common malignant tumor in the department of radiation oncology, as the research object.^[9]^ Radiotherapy for EC has a wide range of radiation and high doses, which can easily lead to myelosuppression.^[10]^ Therefore, it is necessary to guard against the aggravating effect of APDs on thrombocytopenia during radiotherapy. Through retrospective analysis, we have found that the continuous use of APDs during radiotherapy does exacerbate thrombocytopenia but does not increase the incidence of bleeding events. This finding may be due to the timely and sufficient application of platelet-increasing drugs, such as recombinant human interleukin-11 (rhlL-11) and recombinant human thrombopoietin injection (TPIAO). We hope that our research results can provide some reference for radiation oncologists. Of course, more reliable and accurate results need to be derived from further prospective studies.

## 2 Materials and methods

### 2.1 Patients

Patients who underwent radiotherapy for EC in the Third Affiliated Hospital of Soochow University from January 2015 to December 2019 were screened. The inclusion criteria were as follows: patients were diagnosed as EC by pathology; the radiotherapy technique was volumetric modulated arc therapy (VMAT); the radiotherapy mode was conventional fractionated radiotherapy (CFRT); the irradiation range included the esophageal lesion or stump; the prothrombin time (PT) and activated partial thromboplastin time (APTT) before radiotherapy were both within the normal range.

The exclusion criteria were as follows: patients took anticoagulant drugs 1 week before or during radiotherapy; patients received concurrent targeted therapy; patients with distant metastases or other malignant tumors; patients with hyperthyroidism or hypothyroidism; patients with a history of bleeding or coagulopathy; patients with aplastic anemia or idiopathic thrombocytopenia; patients with severe liver or kidney damage (transaminase ≥ 2 times the normal value, or creatinine > 221 μmmol/L); the total number of irradiations is less than 30 times.

This study was approved by the Ethics Committee of the Third Affiliated Hospital of Soochow University and followed the guidelines outlined in the Declaration of Helsinki. All patients signed an informed consent.

### 2.2 Severity of thrombocytopenia and bleeding

The severity of thrombocytopenia is divided into four levels (Table 1): mild thrombocytopenia (platelet concentration is 50 × 10^9^/L to 100 × 10^9^/L), generally without bleeding tendency, radiotherapy and oral APDs can be continued; moderate thrombocytopenia (platelet concentration is 30 × 10^9^/L to 50 × 10^9^/L), with bleeding tendency, radiotherapy and oral APDs are suspended; severe thrombocytopenia (platelet concentration is 10 × 10^9^/L to 30 × 10^9^/L), increased bleeding tendency; extremely severe thrombocytopenia (platelet concentration is less than 10 × 10^9^/L), with hemorrhage in the skin and mucosa, and the risk of cerebral hemorrhage.

**Table 1:** Severity of thrombocytopenia and bleeding.

Severity	Thrombocytopenia	Bleeding
Mild	50 × 10^9^/L to 100 × 10^9^/L	Bleeding gums, skin bleeding or ecchymosis, hematuria on a urine test, bloodshot under the microscope on a sputum test
Moderate	30 × 10^9^/L to 50 × 10^9^/L	Gross hematuria, hemoptysis, hematemesis, hematochezia
Severe	10 × 10^9^/L to 30 × 10^9^/L	Bleeding more than 300 mL at a time
Extremely severe	<10 × 10^9^/L	–

The severity of bleeding is divided into three levels (Table 1): mild bleeding, including bleeding gums, skin bleeding or ecchymosis, hematuria on a urine test, and bloodshot under the microscope on a sputum test; moderate bleeding, including gross hematuria, hemoptysis, hematemesis, and hematochezia; severe bleeding, bleeding more than 300 mL at a time. Once bleeding occurs, the patient's oral APDs is to be suspended.

### 2.3 Prevention and treatment of thrombocytopenia during radiotherapy

During radiotherapy, patients took 8 g of Weixuening granule (a Chinese patent medicine) three times a day to prevent thrombocytopenia. The routine blood test was then checked once a week. If thrombocytopenia occurred, 3.0 mg of rhlL-11 would be injected subcutaneously once a day to increase the platelet concentration. Then, the routine blood test was repeated every three days. If the platelet concentration continued to decrease significantly, 15,000 U of TPIAO would be injected subcutaneously, once a day, until the platelet concentration rose steadily. As TPIAO is expensive in China, the patient's consent is required for use.

### 2.4 Statistical analysis

Comparison of proportions was evaluated by Chi-square (χ^2^) test (or Fisher's exact test, if appropriate). Comparison of averages was evaluated by *t* test. A propensity score-matched (PSM) analysis ratio was performed to eliminate the difference in clinical parameters between the two groups of patients. The matched clinical parameters contained age, sex, tumor location, AJCC stage, nasal feeding ratio, neoadjuvant chemotherapy ratio, concurrent chemotherapy ratio, and postoperative radiotherapy ratio. There is a significant difference when the *P* value is <.05. Statistical analyses were performed using IBM SPSS Statistics 22.0 (SPSS Inc, Chicago, IL).

## 3 Results

### 3.1 Comparison and matching of the clinical parameters of the two groups

Table 2 shows the clinical parameters of the two groups of EC patients that may affect the risk of bleeding. A total of 986 patients were included in the original data set. Of these, 34 patients took APDs during radiotherapy. These patients were assigned to the antiplatelet drug (APD) group, while the other patients were assigned to the control group. After comparing the clinical parameters of the two groups, we found that there were significant differences in the age (*P* = .032), tumor location (*P* = .001), AJCC stage (*P* = .018), and the proportion of postoperative radiotherapy (*P* = .034) between the two groups. To avoid the impact of these differences on the thrombocytopenia and bleeding risk of the two groups during radiotherapy, we used propensity score matching for the data of the two groups. After matching at a 1:1 ratio, the APD group and the control group each retained 31 patients. After comparing the clinical parameters of the matched two groups, we found that there were no significant differences between the two groups in age, sex, tumor location, AJCC stage, nasal feeding ratio, neoadjuvant chemotherapy ratio, concurrent chemotherapy ratio, or postoperative radiotherapy ratio (*P* *>* .05).

**Table 2:** The clinical parameters of PSM cohorts for the control and APD groups.

	Unmatched (complete) dataset			Matched (1:1) dataset		
	Control (%)	APD (%)		Control (%)	APD (%)	
Variable	(n = 952)	(n = 34)	*P*	(n = 31)	(n = 31)	*P*
Age			.032			.767
<60	369	7		8	7	
≥60	583	27		23	24	
Sex			.565			.755
Male	766	26		25	24	
Female	186	8		6	7	
Tumor location			.001			.954
Upper	86	8		8	8	
Middle	668	14		15	14	
Lower	198	12		8	9	
AJCC stage			.018			.796
I	147	4		13	12	
II	418	8				
III	387	22		18	19	
Nasal feeding			.330			1
Yes	50	0		0	0	
No	902	34		31	31	
Neoadjuvant chemotherapy			.710			1
Yes	106	5		5	5	
No	846	29		26	26	
Concurrent chemotherapy			.944			1
Yes	857	31		27	28	
No	95	3		4	3	
Postoperative radiotherapy			.034			.800
Yes	335	18		15	16	
No	617	16		16	15	

AJCC = American Joint Committee on Cancer, APD = antiplatelet drug, PSM = propensity-score matched.

### 3.2 Effects of APD on thrombocytopenia during radiotherapy

The platelet concentration before radiotherapy and the lowest platelet concentration during radiotherapy of the two matched groups were collected (Table 3). By comparing the mean values of platelet concentrations between the two groups, we found that there was no significant difference in platelet concentrations between the two groups before radiotherapy (*P* = .524). However, the lowest platelet concentration during radiotherapy in the APD group was significantly lower than that in the control group (*P* = .033). We classified the lowest platelet concentration. During radiotherapy, 5 patients in the control group and 5 patients in the APD group had moderate thrombocytopenia, with no significant difference (*P* = 1). In the control group and the APD group, 0 patients and 1 patient each experienced severe or extremely severe thrombocytopenia during radiotherapy, with no significant difference (*P* = 1).

**Table 3:** The comparison of thrombocytopenia between the control and APD groups.

Group	Number	PLTc before RT (10^9^/L)	Lowest PLTc during RT (10^9^/L)	≥50 × 10^9^/L	30 × 10^9^/L∼50 × 10^9^/L	<30 × 10^9^/L
Control	31	142.65 ± 33.74	65.71 ± 18.32	26	5	0
APD	31	137.56 ± 28.51	55.65 ± 17.98	25	5	1
*t*/χ^2^		0.642	2.182	0.110	0	–
*P*		0.524	0.033	0.740	1	1

APD = antiplatelet drug, PLTc = platelet concentration, RT = radiotherapy.

### 3.3 Effects of APD on the consumption of platelet-increasing drugs and suspension of radiotherapy

The amount of rhlL-11 and TPIAO consumption during radiotherapy in the two matched groups was collected (Table 4). After comparing the average consumption of the two groups, it was found that the amounts of rhlL-11 and TPIAO used in the APD group were higher than those in the control group (*P* = .001 and *P* = .038). When the platelet concentration was below 50 × 10^9^/L, the patient's radiotherapy was suspended until the count rises above 50 × 10^9^/L stably. Five patients in each of the two groups had radiotherapy suspended because the platelet concentration was below 50 × 10^9^/L (Table 4). Although one patient in the APD group had a platelet count lower than 50 × 10^9^/L, the routine blood test repeated the next day showed that the platelet concentration had risen above 50 × 10^9^/L, so the patient's radiotherapy was not suspended. In addition, there was no significant difference between the two groups in the number of average days of radiotherapy suspension because of thrombocytopenia (*P* = .933).

**Table 4:** The comparison of platelet-raising drugs and RT suspension between the control and APD groups.

		Platelet-raising drugs		RT suspension	
Group	Number	rhIL-11	TPIAO	Patients	Days
Control	31	6.35 ± 2.68	1.23 ± 2.62	5	0.65 ± 1.58
APD	31	9.12 ± 3.47	2.23 ± 2.82	5	0.61 ± 1.43
*t*/χ^2^		3.518	2.167	0	0.085
*P*		0.001	0.038	1	0.933

APD = antiplatelet drug, rhlL-11 = recombinant human interleukin-11, RT = radiotherapy, TPIAO = recombinant human thrombopoietin injection.

### 3.4 Effect of APD on bleeding during radiotherapy

Table 5 shows the bleeding in the two groups of patients during radiotherapy. In the control group, two patients experienced moderate bleeding. In the APD group, one patient experienced minor bleeding and one patient experienced moderate bleeding. In both groups, no patients terminated radiotherapy or died because of bleeding. There was no significant difference in the incidence of bleeding between the two groups (*P* = .605).

**Table 5:** The comparison of bleeding between the control and APD groups.

Group	Number	Bleeding	Mild bleeding	Moderate bleeding	Severe bleeding
Control	31	2	0	2	0
APD	31	2	1	1	0
χ^2^		0.267	-	0	–
*P*		0.605	1	1	1

APD = antiplatelet drug.

## 4 Discussion

Our results show that APDs have no significant effect on the platelet concentration of EC patients before radiotherapy. This confirms that thrombocytopenia is indeed not a common side effect of APDs. However, continuous oral APDs during radiotherapy can cause a further decrease in platelet concentration, which may be related to the pharmacology of APDs.^[11]^ Clopidogrel is a commonly used APD in the APD group. The activated clopidogrel selectively and irreversibly inhibits the binding of adenosine diphosphate (ADP) to the platelet receptor P2Y12 and the activation of the ADP-mediated glycoprotein GPIIIb/IIIa complex to inhibit platelet aggregation^[12]^. The combination of clopidogrel and P2Y12 inhibits platelet aggregation while also shortening the lifespan of platelets.^[12]^ During radiotherapy, radiation kills the sensitive megakaryocytes in the bone marrow, resulting in a decrease in mature megakaryocytes and peripheral platelets.^[13]^ Therefore, continuous oral clopidogrel during radiotherapy will simultaneously inhibit platelet production and promote platelet death, which may lead to more severe thrombocytopenia.

In addition, our results also show that oral APDs do not increase the proportion of severe thrombocytopenia and the incidence of bleeding events in EC patients during radiotherapy. Severe bleeding can cause death or the termination of treatment.^[14]^ Fortunately, no severe bleeding events were observed in the included 62 EC patients. This is probably because the tumor lesions of most patients did not invade large blood vessels and our timely intervention for thrombocytopenia. Regarding whether bleeding during radiotherapy will affect the long-term outcome of EC patients, because of the small number and short follow-up time of patients included in this study, we cannot yet draw a definitive conclusion. Moreover, there was no difference between the two groups of patients in the suspension of radiotherapy because of thrombocytopenia. However, these achievements are based on the timely and adequate application of platelet-increasing drugs. On average, patients in the APD group used approximately 9 mg of rhIL-11 and 15,000 U of TPIAO more than patients in the control group. If economic conditions allow, we still recommend that patients who have long-term oral APDs continue to take APDs during radiotherapy. This will not affect the patient's radiotherapy efficacy, nor will it increase the risk of cardio-cerebral vascular infarction.

The most commonly used platelet-increasing drugs in our hospital are rhlL-11 and TPIAO. In addition, rhlL-11 is a platelet-promoting growth factor produced by genetic recombination technology, which can directly stimulate the proliferation of bone marrow hematopoietic stem cells and megakaryocyte progenitor cells, induce the maturation and differentiation of megakaryocytes, and increase the production of platelets in the body.^[15]^ Preclinical studies have shown that the mature megakaryocytes after the application of rhlL-11 in vivo are completely normal in ultrastructure, and the morphology, function and lifespan of platelets produced are also normal.^[16]^ Thrombopoietin (TPO) is an endogenous cytokine that stimulates the growth and differentiation of megakaryocytes.^[17]^ It has a stimulating effect on all stages of megakaryocyte production, including the proliferation of precursor cells and the development and maturation of polyploid megakaryocytes.^[18]^ TPIAO is a purified full-length glycosylated thrombopoietin expressed by Chinese hamster ovary cells using genetic recombination technology.^[19]^ It has a similar pharmacological effect of increasing platelets with endogenous thrombopoietin. Compared with rhlL-11, TPIAO has a stronger platelet-increasing effect and less side effects, but it is much more expensive, so it is generally used as a remedy for rhlL-11. There is currently no research showing that APDs have drug interactions with rhlL-11 or TPIAO. Therefore, it was ruled out that the APD group used more platelet-increasing drugs during radiotherapy than the control group because of the inhibition of APDs on platelet-increasing drugs.

EC is one of the common diseases in our department. As radiation oncologists, we are alert to the occurrence of severe bleeding in EC patients during radiotherapy, because the blood supply around the esophagus is abundant and the tumor lesions easily invade the large blood vessels. Once this type of bleeding occurs, it is difficult to stop, and the mortality rate is extremely high. Therefore, clinically, taking APDs is very important in the medical history of EC patients. Because of the lack of related research, it is uncertain whether APDs will increase the risk of bleeding during radiotherapy, so we often advise patients to stop taking APDs during radiotherapy. However, because the duration of CFRT for most ECs is 5 to 7 weeks,^[10]^ stopping oral APDs from the beginning to end during radiotherapy may lead to cardiovascular and cerebrovascular thrombosis.

Through this retrospective analysis, we confirmed that APDs can lead to a further reduction in platelets during radiotherapy, which may increase the risk of bleeding and reduce the efficacy of radiotherapy, but timely and adequate application of platelet-increasing drugs can prevent these adverse events. In other words, the further reduction of platelets caused by APDs is controllable, which is of great significance for ensuring the safety of malignant tumor patients with cardiovascular and cerebrovascular diseases during radiotherapy. Furthermore, we found that because patients in the APD group took timely and adequate platelet-increasing drugs, only a small percentage of these patients temporarily suspended oral APDs. Therefore, platelet-increasing drugs can maintain the balance between bleeding and thrombosis during radiotherapy. As long as the platelet count is closely monitored and platelet-increasing drugs are applied in time and in sufficient quantities, APDs can be taken continuously during radiotherapy.

Although this study is a retrospective study, we have eliminated the differences caused by many confounding factors through the method of PSM analysis. This method improves the reliability of the results. However, this study also has shortcomings. PSM analysis, on the other hand, reduced the sample size, and only 62 patients of 986 patients were included in the matched data set. APDs contain a variety of drugs, and their pharmacology, side effects, and drug interactions are different. It is better to perform subgroup analysis by the types of APDs. Unfortunately, because of the limitation of sample size, we were unable to conduct subgroup analysis. In addition, the types of chemotherapy drugs used by these patients are also different, which has a significant inhibitory effect on platelet concentration, and subgroup analysis is also required.

In conclusion, continuous oral APDs during radiotherapy will lead to a further decrease in platelet concentration. Timely and adequate application of platelet-increasing drugs, such as rhlL-11 and TPIAO, can avoid the increased risk of bleeding and the reduced efficacy of radiotherapy caused by oral APDs. Prospective controlled studies with larger samples are needed to further confirm our results.

## Author contributions

**Conceptualization:** Wendong Gu, Qilin Li.

**Data curation:** Dan Xi, Wenjie Jiang, Yingjie Shao, Xing Song, Yuan Chen, Mengjiao Liu.

**Formal analysis:** Dan Xi, Wenjie Jiang.

**Software:** Dan Xi, Xing Song, Mengjiao Liu.

**Validation:** Dan Xi, Wendong Gu, Qilin Li.

**Writing – original draft:** Dan Xi.

## Abbreviations

Abbreviations: ADP = adenosine diphosphate, APD = antiplatelet drug, APDs = oral antiplatelet drugs, APTT = activated partial thromboplastin time, CFRT = conventional fractionated radiotherapy, EC = esophageal cancer, PSM = propensity-score matched, PT = prothrombin time, rhlL-11 = recombinant human interleukin-11, TPIAO = recombinant human thrombopoietin injection, TPO = thrombopoietin, VMAT = volumetric modulated arc therapy.
